# Seroprevalence of COVID-19 in Riyadh city during the early increase of COVID-19 infections in Saudi Arabia, June 2020

**DOI:** 10.1016/j.sjbs.2022.103282

**Published:** 2022-04-22

**Authors:** Mohammed W. Alenazi, Abdullah Algaisi, Hosam M. Zowawi, Omar Aldibasi, Anwar M. Hashem, Naif Khalaf Alharbi

**Affiliations:** a King Abdullah International Medical Research Center, Riyadh, Saudi Arabia; b King Saud bin Abdulaziz University for Health Sciences, Riyadh, Saudi Arabia; c Department of Medical Laboratories Technology, College of Applied Medical Sciences, Medical Research Center, Jazan University, Jazan 45142, Saudi Arabia; d Department of Medical Microbiology and Parasitology, Faculty of Medicine, King Abdulaziz University, Jeddah, Saudi Arabia; e Vaccines and Immunotherapy Unit, King Fahd Medical Research Center, King Abdulaziz University, Jeddah, Saudi Arabia

**Keywords:** Seroprevalence, COVID-19, SARS-CoV-2, ELISA, Blood donors

## Abstract

Severe acute respiratory syndrome coronavirus (SARS-CoV-2) emerged in December 2019 and caused a global pandemic of the Coronavirus Disease 2019 (COVID-19). More than 170 million cases have been reported worldwide with mortality rate of 1–3%. The detection of SARS-CoV-2 by molecular testing is limited to acute infections, therefore serological studies provide a better estimation of the virus spread in a population. This study aims to evaluate the seroprevalence of SARS-CoV-2 in the major city of Riyadh, Saudi Arabia during the sharp increase of the pandemic, in June 2020. Serum samples from non-COVID patients (n = 432), patients visiting hospitals for other complications and confirmed negative for COVID-19, and healthy blood donors (n = 350) were collected and evaluated using an in-house enzyme-linked immunosorbent assay (ELISA). The overall percentage of positive samples was 7.80% in the combined two populations (n = 782). The seroprevalence was lower in the blood donors (6%) than non-COVID-19 patients (9.25%), p = 0.0004. This seroprevalence rate is higher than the documented cases, indicating asymptomatic or mild unreported COVID-19 infections in these two populations. This warrants further national sero-surveys and highlights the importance of real-time serological surveillance during pandemics.

## 1 Introduction

Human coronaviruses (CoVs) were first recognized in 1960s as main infectious agents of the upper respiratory tract (Kahn and McIntosh, 2005). Several types of human and animal CoVs that were identified in the world in the last two decades represent a serious issue to public health including the severe acute respiratory syndrome coronavirus (SARS-CoV), which appeared from 2002 to 2003. In 2012, a new epidemic was identified for the first time in Saudi Arabia caused by a novel coronavirus known as the Middle East respiratory syndrome coronavirus (MERS-CoV) (Cascella et al., 2020). MERS-CoV infects dromedary camels and transmits into humans as a zoonotic virus. In Saudi Arabia, around 2500 cases of MERS were reported with 800 deaths, 35% fatality rate (WHO, 2019). In 2019, a novel coronavirus emerged in Wuhan, China and named SARS-CoV-2. SARS-CoV-2 causes a severe acute respiratory syndrome, termed Coronavirus Disease 2019 (COVID-19). Symptoms of COVID-19 vary from mild respiratory infection to severe pneumonia, and death. In March 2020, the World Health Organization (WHO) announced COVID-19 as a global pandemic (Song et al., 2020, WHO, 2020, Yuen et al., 2020).

High numbers of infections across the globe had been reported in the first weeks of the pandemic, causing a huge pressure on hospitals and healthcare systems, which encouraged countries to take further infection control precautions. In June 2020, the number of infections in Europe and the United States of America exceeded 2.5 and 4 millions, respectively (WHO, 2020). Therefore, seroepidemiology is a key research area to understand the public health burden of the pandemic as well as to shape policies and efforts for vaccine development and countermeasures. While antibodies against SARS-COV-2 could be detected in the first seven days of the infection, IgM and IgG antibodies can be detected in blood at 10–12 days and 12 to 21 days post-onset in most cases, respectively (Guo et al., 2020, Lou et al., 2020, Young et al., 2020). Although detection of SARS-CoV-2 viral RNA by reverse transcriptase-polymerase chain reaction (RT-PCR) is the gold standard method for diagnosis (World Health Organization, 2020), detection of serum anti-SARS-CoV-2 antibodies can provide a practical method of understanding the COVID-19 prevalence as some cases remain asymptomatic and are not detected by RT-PCR testing (Chan et al., 2020, Li et al., 2020, Rothe et al., 2020). Therefore, it is expected that seroprevalence rate is higher than the reported cases of COVID-19 as shown by a number of serological studies across the globe (Pollán et al., 2020, Poustchi et al., 2020, Rostami et al., 2020, SeroTracker., n.d.).

In Saudi Arabia, the accumulative number of COVID-19 cases were ∼40,000 in early May 2020, increased to the peak at ∼157,000 cases on 20th of June 2020 before starting to decline from  >4000 case a day in mid-June to ∼500 case a day in September 2020, which was the first wave of the pandemic in the country. In this study, we aimed to assess SARS-CoV-2 seroprevalence in the capital city of Riyadh, which has a population of ∼7 million, in samples collected from blood donors and in- and out-patients who are visiting hospitals for other complications but they were confirmed negative for COVID-19 (reffered to as non-COVID-19). The samples were collected in the first two weeks of June, the period where Saudi Arabia experienced the highest reported cases, in 2020.

## 2 Materials and methods

### 2.1 Samples

A total of 782 serum samples were collected in the first two weeks of June 2020 from two different cohorts at King Abdulaziz Medical City (KAMC), Riyadh. The first cohort was residual serum samples from non-COVID patients (n = 432) from the clinical Biochemistry Lab. Non-COVID-19 patients were defined as any patients visiting hospitals for routine or emergency Biochemistry blood tests; these patients were required to do nasopharyngeal sample testing for SARS-CoV-2 RT-PCR. All patients were confirmed negative as documented in their medical records, and considered non-COVID-19 patients. The second cohort (n = 350) was from healthy blood donors collected from the Blood Bank; the donors were routinely screened for SARS-CoV-19 by RT-PCR on nasopharyngeal swabs and confirmed negative. All serum samples were transferred to King Abdullah International Medical Research Center (KAIMRC) and tested by an in-house enzyme-linked immunosorbent assay (ELISA).

### 2.2 Recombinant s proteins

Three different SARS-CoV-2 recombinant S1 proteins were chosen for testing and comparison including proteins from Native Antigen, UK (Lot No. 20042111), Sinobiological Ltd, China (Cat. No. 40591-v08B1), and GenScript, USA (Cat. No. Z03501).

### 2.3 Elisa

An in-house enzyme-linked immunosorbent assay (ELISA) was used to detect IgG antibodies against SARS-COV-2 in serum samples. The ELISA was performed following the previously reported procedure (Degnah et al., 2020, Hodgson et al., 2014). Briefly, Nunc MaxiSorp 96-well ELISA microplates (Thermo Fisher, Waltham, MA) were coated with recombinant S1 subunit of the SARS-CoV-2 Spike protein (Sinobiological Ltd, China (Cat. No. 40591-v08B1) at a concentration of 1 μg/ml. The plates were incubated at room temperature (RT) overnight. Next day, the plates were washed six times with phosphate buffered saline (PBS) with 0.5% Tween20 (PBS-T) using automated microplate washer (Molecular Devices, San Jose, CA). The plate wells were blocked by 100 μl washing buffer containing 10% skimmed milk (blocking buffer) for one hour at RT. The serum samples were diluted 1:100 in PBS-T, and 50 μl of each diluted sample were added into duplicate wells then incubated for two hours at RT. Then, 50 μl of 1:1000 diluted alkaline phosphatase labeled goat anti-human IgG secondary antibody (Thermo Fisher, Waltham, MA) were added and incubated for one hour at RT. The plates were then washed six times, and the pnitrophenylphosphate (PNPP) substrate dissolved in diethanolamine buffer and deionized water was added. The optical density (OD_405_) was measured using a microplate reader (Molecular Devices, San Jose, CA). The cut-off value was set as the average of the negative control serum samples plus three times of standard deviation. The Negative control samples were sera collected before the COVID-19 pandemic, and the positive control samples were from confirmed recovered COVID-19 cases. The same control samples were aliquoted and used in every ELISA run.

### 2.4 Optimization of ELISA

In order to optimize the in-house ELISA, the three commercially available spike S1 proteins were tested and compared. Eight serum samples that have been previously tested and showed various endpoint titres ranging from strong positive to negative (unpublished) were used in the ELISA. This evaluation showed that Sinobiological and NativeAntigen were able to distinguish negative sera from positive sera with similar rank of positive, borderline positive, and negative as expected and as previously determined by endpoint titre ELISA or neutralization assays. While both groups of proteins had lower background signal, samples were clearly distinguished when using S1 protein from Sinobiological, followed by NativeAntigen (Fig. S1, supplementary file), suggesting that this protein is the most suitable for testing, consistent with previous studies (Algaissi et al., 2020, Tan et al., 2020).

## 3 Results

### 3.1 Seroprevalence of SARS-CoV-2 in Riyadh

**Fig. 1:**
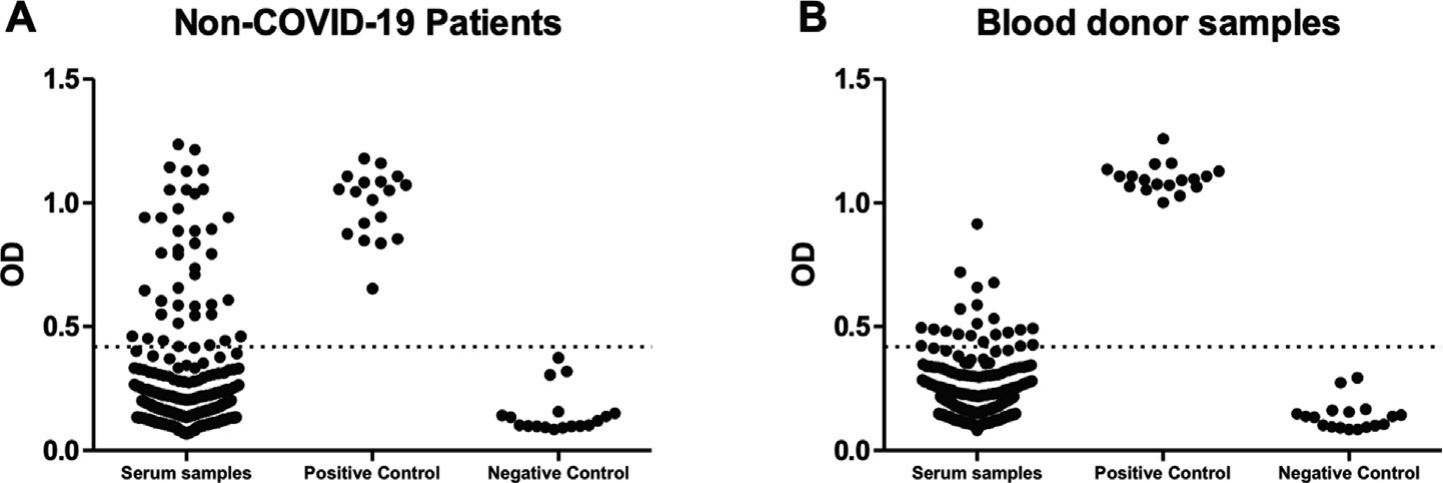
**Seropositivity of sera from blood donors and non-COVID-19 patients from Riyadh, Saudi Arabia, June 2020.** In-house ELISA for anti-SARS-CoV-2 spike IgG antibodies for serum samples from non-COVID-19 patients (**A**) and blood donors (**B**) were evaluated along with positive and negative control samples. The in-house ELISA's cut-off value was calculated as the average of negative controls plus three times the standard deviation.

The seroprevalence of COVID-19 in healthy individuals who donated blood (n = 350) and in patients who visited the hospital for non-COVID-19 clinical issues (n = 432) was determined. Out of the 432 samples from non-COVID-19 patients, 40 samples were positive giving a seroprevalence rate of 9.25%. On the other hand, 6% (21/350) of the serum samples from blood donors were positive (Fig. 1). The seroprevalence rate in non-COVID-19 patients was significantly higher than that in blood donors in Riyadh during the peak of COVID-19 cases in Saudi Arabia (P = 0.0004, by Chi-square Test). This indicates the susceptibility of individuals with other medical issues or comorbidities.

## 4 Discussion

This study reports the seroprevalence of SARS-CoV-2 in Riyadh city during the early rise of COVID-19 cases in Saudi Arabia in June 2020. COVID-19 cases in Saudi Arabia were ∼90,000 in early June, and peaked to ∼157,000 cases on 20th of June 2020 before starting to decline (Saudi Ministry of Health, 2021). This study targeted healthy population by sampling blood donors and co-morbid/unhealthy populations (people visiting hospital for routine emergency diagnosis, but they are not COVID-19 cases). Overall percentage of positive samples was 7.80% in the combined two populations (n = 782). The seroprevalence was lower in the blood donors (6%) than non-COVID-19 patients (9.25%), indicating asymptomatic or mild unreported COVID-19 infections in these two populations. Based on these data, rate of seropositivity in blood donor is expected to be lower than other populations especially that none of the donors reported any history of COVID-19 infection or exposure to COVID-19 cases, as part of screening process for blood donation. In addition, it is possible that co-morbid patients would be more prone to contract infections, hence increased seroprevalence particularly that comorbidities such as hypertension and diabetes have been reported as risk factors for COVID-19 (Cook, 2020, Das et al., 2020, Du et al., 2021, Ejaz et al., 2020, Wang et al., 2020).

An early national serosurvey of blood donors from 24 major blood banks in Saudi Arabia showed seroprevalence of 1.4% (12/837) in samples collected at the end of May 2020 (Banjar et al., 2021). Interestingly, seropositivity in Riyadh from this study was found to be 0.0% (Banjar et al., 2021). Other local studies reported varying rates of seroprevalence among blood donors in different cities within the country that ranged from 0.0% in Jeddah during the early months of the pandemic from 1 January to 31 May 2020 (Alandijany et al., 2021) to 19% in the city of Madinah from mid-May to mid-July 2020 (Mahallawi and Al-Zalabani, 2021). In the current study we found that 6% of blood donors were seropositive which is higher than previously reported (Banjar et al., 2021). These differences could be attributed to differences in methods used, timing of the studies and transmission patterns overtime or between regions. During this study period, Riyadh region had an incident rate of 0.8% compared to Madinah which had a rate of 1.27%. Another possibility is that some regions had lower number of COVID-19 diagnostic tests, resulting in lower number of reported cases and lower incident rate. The COVID-19 testing capacity in Saudi Arabia was excellent and distributed widely across the kingdom (Saudi Ministry of Health, 2021), but we speculate that people in some regions with mild disease might not have sought COVID-19 testing.

While testing in the current study was performed by in- house ELISA without neutralization assay, we used the best commercially available recombinant antigen which has been used in many seroepidemiological studies globally. In addition, we used known positive and negative control samples that have been tested in endpoint titre ELISA as well as neutralization assay to validate our assay. To improve subsequent studies, larger number of samples from different hospitals and/or regions are required to obtain more accurate estimation of the true seroprevalence of COVID-19 in the country, which was not logistically possible for the current work. This study only focused on blood donors and non-COVID-19 patients (who visited the hospital) during the first 2 weeks of June 2020. Thus, future studies would also require repeated testing over extended period of time to determine the actual prevalence of COVID-19. However, this study is the only report on the seroprevalence in blood donors in Saudi Arabia just at the time were COVID-19 started to rise in the country in early June 2020. Therefore, it could provide a reference point for future serological surveys. Overall, serological surveys, if they are conducted on real-time and throughout the months of a pandemic can be useful predictive measure to determine the extent of asymptomatic transmission. Thus, their results would guide public health interventions and infection control measures on a timely manner that would contribute into controlling pandemics.

## Author contribution

MWA and HMZ collected samples. MWA and NKA ran lab experiments. AA, AMH, and OA analysed the data. MWA and NKA wrote the manuscript. NKA obtained fund and supervized the study. All authors read, revised and approved the manuscript.

## Funding

This study was funded by the Saudi Ministry of Heath; COVID-19 Research grant number: 514.

## Declaration of Competing Interest

The authors declare that they have no known competing financial interests or personal relationships that could have appeared to influence the work reported in this paper.
