# Supplementary material for: Seroprevalence of COVID-19 in Riyadh city during the early increase of COVID-19 infections in Saudi Arabia, June 2020
Source: Saudi J Biol Sci. 2022 Apr 22;29(6):103282. doi: 10.1016/j.sjbs.2022.103282 (PMC9027288; doi:10.1016/j.sjbs.2022.103282)
Supplement: Supplementary data 1 [file mmc1.pdf]

Supplementary File

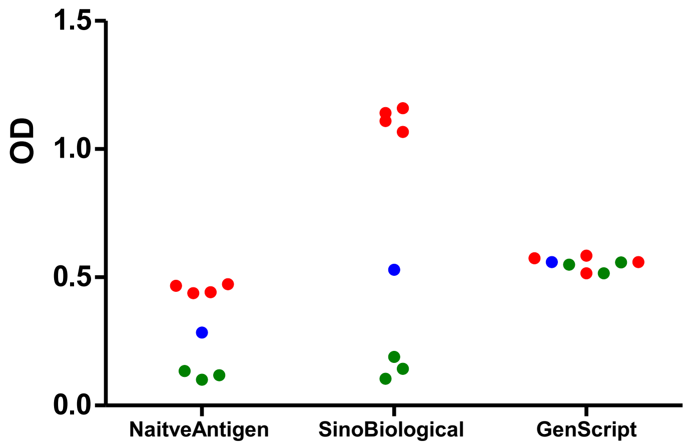

Figure S1: Testing of different recombinant spike S1 proteins in ELISA
Three commercially available recombinant spike S1 proteins of SARS-CoV-2 were evaluated using in-house ELISA; referred to by their manufacturers: Sinobiological, GenScript, NativeAntigen. The proteins were tested against eight serum samples that are known to be negative (green), borderline positive (blue), or positive (red). OD: Optical density.
